# Validation and Determination of Physical Activity Intensity GT3X+ Cut-Points in Children and Adolescents with Physical Disabilities: Preliminary Results in a Cerebral Palsy Population

**DOI:** 10.3390/children10030475

**Published:** 2023-02-27

**Authors:** Carmen Matey-Rodríguez, Susana López-Ortiz, Saúl Peñín-Grandes, José Pinto-Fraga, Pedro L. Valenzuela, Mónica Pico, Carmen Fiuza-Luces, Simone Lista, Alejandro Lucia, Alejandro Santos-Lozano

**Affiliations:** 1 i+HeALTH Strategic Research Group, Department of Health Sciences, Miguel de Cervantes European University (UEMC), 47012 Valladolid, Spain; 2 Research Institute of the Hospital 12 de Octubre (‘imas12’), 28041 Madrid, Spain; 3 Department of Systems Biology, University of Alcalá, 28871 Madrid, Spain; 4 Faculty of Sport Sciences, European University of Madrid, Villaviciosa de Odón, 28670 Madrid, Spain

**Keywords:** pediatrics, energy expenditure, motor impairment, motor disorder

## Abstract

Background: Children and adolescents with disabilities engage in low levels of moderate-to-vigorous intensity physical activity (MVPA), which may create the onset of a sedentary lifestyle. In light of this, MVPA levels must be quantified with a valid tool such as accelerometry. This study aimed to: (i) analyze the accuracy of Evenson cut-points by estimating MVPA and sedentary behavior (SB) in children and adolescents with disabilities; (ii) define new equations to estimate energy expenditure (EE) with the GT3X+ accelerometer in this population and particularly in those with cerebral palsy (CP); (iii) define specific GT3X+ cut-points to estimate MVPA in those with CP. Methods: A total of 23 children and adolescents with disabilities (10 ± 3 years; 44%females) participated in the study. GT3X+-counts and oxygen uptake (VO_2_) were measured in four laboratory walking conditions. Results: (i) Evenson cut-points were accurate; (ii) new equations were defined to effectively predict EE; (iii) specific GT3X+ cut-points (VM ≥ 702 counts·min^−1^; Y-Axis ≥ 360 counts·min^−1^) were defined for estimating MVPA levels in children and adolescents with CP. Conclusions: The use of specific cut-points for ActiGraph GT3X+ seems to be accurate to estimate MVPA levels in children and adolescents with disabilities and, particularly, in those with CP, at least in laboratory conditions.

## 1. Introduction

Children and adolescents, including those with disabilities, should participate in regular physical activity (PA) to enhance health and wellbeing [1]. In fact, the World Health Organization (WHO) released, in 2020, updated global guidelines on physical activity and sedentary behavior for children and adolescents aged 5–17 years. In particular, children and adolescents are recommended to engage in an aerobic activity of moderate-to-vigorous intensity PA (MVPA) for at least 60 min each day. They also need to perform an aerobic activity of vigorous intensity, as well as strengthening exercises, at least three days a week. Finally, they should aim to reduce sedentary behavior (SB), especially recreational screen activities [2]. However, children and adolescents with disabilities not only participate in less MVPA than their typically developing peers [3], but become less physically active with age and with the development of health conditions, which can lead to deconditioning and the start of a sedentary lifestyle perpetuation cycle [4,5].

The available strategies to quantify PA in young people include observation, parental self-report, and accelerometry [6,7]. Accelerometer-based activity monitoring is a valid indicator of daily PA in both children and adolescents with and without disabilities because accelerometers estimate the volume and intensity of PA and the pattern of both active and sedentary behavior [8]. Currently, ActiGraph accelerometers are one of the most widely used wearable devices for the quantification of PA and SB [9,10]. However, specific metabolic equations and cut-points—intensity-based thresholds [11]—commonly defined according to metabolic equivalents of task (METs) [12], namely moderate intensity: 3.00–5.99 METs; vigorous intensity: 6.00–8.99 METs; and very vigorous intensity: ≥9 METs—are needed for each model of accelerometer and population to ensure an accurate PA pattern estimation [13].

Clinicians typically work with children and adolescents who have a wide range of disabilities [14]; however, the use of different equations and cut-points for each individual may make their professional activity extremely demanding, especially when the analysis of accelerometer data requires time and experience to attain data of high quality [15]. Evenson’s cut-points (i.e., SB: <25 counts·15 s^−1^; light PA: 26–573 counts·15 s^−1^; MVPA: >574 counts·15 s^−1^ [6]) are commonly used to estimate PA levels in children [16]. However, Evenson’s cut-points were developed with an older generation of ActiGraph devices (i.e., AM764, MTI). Thus, using Evenson’s cut-points with the newest generation of device (GT3X/+) may not be appropriate because devices produce different activity counts for a given acceleration [17]. Indeed, Evenson’s cut-points provide a moderately accurate estimation of MVPA in children and adolescents with cerebral palsy (CP)—the most common disability in childhood [18]—through the GT3X+ accelerometer [19]. However, to the best of our knowledge, the accuracy of the Evenson cut-points has not been tested in a cohort of children and adolescents with physical heterogenous disabilities who can walk with or without devices (Gross Motor Function Classification System (GMFCS) levels I–III) [20,21]. Furthermore, based on the currently available scientific literature, there are only two studies defining specific MVPA algorithms for children and adolescents with CP for the GT3X+ ActiGraph accelerometer, and these involve complex analysis procedures [22] replacing the traditional cut-point values. Trost and colleagues (2016) [23] defined decision trees for detection of PA intensities in young children with CP, and Goodlich and colleagues (2020) [22] investigated the accuracy of machine learning models to quantify PA intensities in children with CP. Finally, none of the available equations is able to accurately predict the energy expenditure (EE) during PA in children with CP [19].

For these reasons, the aims of our study were: (i) to analyze the accuracy of Evenson cut-points by estimating MVPA and SB in children and adolescents with physical heterogeneous disabilities who can walk with or without devices; (ii) to define new equations to estimate EE with the GT3X+ in children and adolescents with physical heterogeneous disabilities (as well as those with CP) who can walk with or without devices; and (iii) to define specific GT3X+ cut-points in order to estimate MVPA in children and adolescents with CP.

## 2. Materials and Methods

### 2.1. Study Design

A cross-sectional study was conducted at the Exercise Physiology Laboratory of the Miguel de Cervantes European University (Universidad Europea Miguel de Cervantes (UEMC, Valladolid, Spain)). The research team consisted of PhD sports scientists, PhD M.D., PhD O.D., PhD molecular biologist, physiotherapists, and sports scientists. The study was performed according to the declaration of Helsinki and approved by the University’s Human Ethics Committee (Miguel de Cervantes European University, protocol code 6586).

### 2.2. Study Participants

Participants were recruited in Valladolid, Spain, between May 2022 and June 2022. The information of the study was provided to the physiotherapists that worked in primary and secondary schools in Valladolid and Palencia (Spain). They sent the information to the tutors of the eligible children and adolescents with the contact details of the study investigators. Interested legal tutors of children and adolescents contacted the researchers. Inclusion criteria were represented by children and adolescents aged 5 to 18 years with physical disabilities (confirmed by medical diagnosis) that were able to walk with and without technical aids (levels I-III of GMFCS [20,21]). Children and adolescents were ineligible if they: (i) had undergone orthopedic surgery within the past 6 months, (ii) had lower extremity botulinum toxin injections within the last 3 months, (iii) presented a recent musculoskeletal injury or medical condition limiting their ability to complete the PA assessment protocol, and (iv) did not present verbal comprehension allowing them to properly understand the instructions of the tests. Participants were also excluded if they had any other contraindications to exercise.

Tutors gave signed informed consent and children gave their verbal assent to participation in the study.

### 2.3. Study Procedure

The study consisted of one visit. Upon arrival at the laboratory, all participants underwent a standardized familiarization visit, prior to data collection, to meet the laboratory personnel and ensure that they were comfortable, properly understood the instructions, and were ready to undertake the study measurements. After taking the anthropometric measures (in light clothing and without footwear), participants underwent the test protocol for recording energy expenditure measurements.

The test protocol followed the procedure established by Clanchy and colleagues (2011) and O’Neil and colleagues (2014) for a similar purpose and population [24,25]. The protocol consisted of four conditions (of 4 min duration each) based on behavioral verbal clues: (i) resting and (ii) treadmill (Pulsar, h/p/cosmos, Nussdorf-Traunstein, Germany) walking at a comfortable pace (clue: “comfortable normal speed, like you do with friends at school or parents at the street”), (iii) treadmill walking at brisk-paced walking (clue: “like you are hurrying to get back to class after the bell has rung”), and (iv) treadmill walking at fast-paced walking (clue: “as fast as you possibly can without falling over or running”). Between walking conditions, participants were asked to rest for a maximum time period of 10 min until they had returned to their resting heart rate (HR) and oxygen values. The protocol was supervised by a physiotherapist and by a sports scientist. Because of the nature of the protocol, researchers were not blinded to the condition. At the end of the test protocol, all participants were monitored until oxygen consumption and HR returned to the resting values.

### 2.4. Outcomes Measures

Physical activity intensity was measured by portable indirect calorimeter and reported as METs. During the test protocol, each participant simultaneously wore a GT3X+ unit and a portable indirect calorimeter. The characteristics of these instruments are detailed below.

#### 2.4.1. Accelerometer

Three GT3X+ units were updated with the v1.9.2 Firmware version. All units were initialized via a computer interface to collect data at a sampling frequency of 30 Hz (the acceleration signal related to human movement is primarily found below 10 H [26]) with the normal data filter selected. Each participant wore one unit (randomly chosen) positioned securely on the participant’s right hip using an elastic belt. Two researchers (SLO, ASL) checked the position of the monitor before and after each condition (see details below).

The ActiGraph GT3X+ monitor device (ActiGraph, Pensacola, FL, USA) is lightweight (19 g), compact (4.6 × 3.3 × 1.5 cm), and has a rechargeable lithium polymer battery [27]. GT3X+ uses a solid-state tri-axial accelerometer to collect motion data on the 3 axes (i.e., vertical (Y), horizontal right–left (X), and horizontal front–back axis (Z)); additionally, the vector magnitude (VM) may be computed by ActiGraph software. The device measures and records time-varying accelerations ranging from -6 to 6 Gs. The accelerometer output is digitized by a 12-bit analog to digital convertor (ADC) at a rate up to 100 Hz. After being digitized, the signal is passed through a digital filter, which limits the frequency range of the accelerometer to 0.25–2.5 Hz. Each sample is averaged over an ‘epoch’, and the ActiGraph output is given in ‘counts’. The counts obtained during each time period depend on the amplitude and frequency of movements during that time period [28].

#### 2.4.2. Portable Indirect Calorimeter

Oxygen uptake (VO_2_) was measured continuously ‘breath-by-breath’ during each condition by using indirect calorimetry (Cortex, Metalyzer 3B, Leipzig, Germany). The metabolic cart was calibrated with a known gas mixture (16% O_2_ and 5% CO_2_) and volume prior to testing each participant [29]. Occasional errant breaths (e.g., due to coughing, swallowing, or talking) were deleted from the data set when exceeding 3 standard deviations around the local mean, the latter being defined as the average of 2 following and 2 preceding sampling intervals [30]. HR was measured by connecting a Polar H10 sensor chest-strap device (Polar Electro Oy, Kempele, Finland) to the gas analyzer.

### 2.5. Data Reduction

Data from minutes 2–4 were used in the analysis to ensure that a “steady state oxygen consumption” had been achieved. For each participant, a steady state was confirmed by inspection of HR and VO_2_ values [31,32]. METs (estimates of PA intensity) were calculated individually (mean VO_2_/resting metabolic rate) [13,24]. Activity counts were obtained by averaging the activity counts of the two last minutes of Y-Axis and VM. Accelerometers and portable indirect calorimeter were time synchronized using an internal computer clock.

### 2.6. Data Analysis

Statistical analyses were performed by a PhD sports scientist with a master’s degree in Biostatistics and with research experience (ASL) using Stata 14.0 (StataCorp, College Station, TX, USA), PRISM 8 (GraphPad, San Diego, CA, USA), MedCalc Software Ltd. (MedCalc, Ostend, Belgium). The researcher was blinded to the activities from which the data came. Data are presented as mean ± standard deviation (SD) unless stated otherwise. The significance level was set at *p* ≤ 0.05.

The statistical procedures used for each of the study objectives are described below.

#### 2.6.1. Study Objective (i): To Analyze the Accuracy of Evenson Cut-Points Estimating MVPA and SB in Children and Adolescents with Heterogeneous Disabilities Who Can Walk with or without Devices

Sensitivity, specificity, and area under the receiver operating characteristic curve (ROC-AUC value) [33] were calculated to assess the ability of the Evenson cut-points to accurately classify the PA intensity level of the participants. AUC values of 0.90 were defined as excellent accuracy, 0.80–0.89 as good, 0.70–0.79 as fair, and <0.7 as poor [24,34].

#### 2.6.2. Study Objective (ii): To Define New Equations to Estimate EE with the GT3X+ in Children and Adolescents with Heterogeneous Disabilities or CP Who Can Walk with or without Devices 

To determine the new equations in children and adolescents with heterogeneous disabilities or CP who can walk with or without devices, random-coefficient models were used to explore the relationship between METs and GT3X counts·min^−1^ (from Y-Axis and VM) over the four mentioned conditions, while accounting for the dependence among repeated measurements taken on the same child or adolescent (see details elsewhere [35]). The sex, age, weight, and height were introduced in the model as covariables, but in the reported equations we only included the covariables that contributed significantly to the fit the model. A leave-one-out cross-validation was performed to assess the model accuracy.

Also, the accuracy of the new proposed equations was examined by Bland–Altman plot with multiple measurements per individual [36]; bias and 95% limits of agreement (LOA) for each plot were also calculated. The MOVER method was used to estimate the confidence intervals of the limits of agreement [37]. The association between the difference and the magnitude of the measurement (i.e., heteroscedasticity) was examined by regression analysis, entering the difference between the EE measured and the EE estimated using the EE (METs) of the proposed new equation as the dependent variable and the averaged value [(indirect calorimetry + EE estimated)/2] as the independent variable [38].

#### 2.6.3. Study Objective (iii): To Define GT3X+ Cut-Points to Estimate MVPA in Children and Adolescents with CP

ROC curve analysis was computed to identify the count threshold that maximized sensitivity and specificity for discriminating MVPA in children and adolescents with CP. Also, AUCs were calculated and, as previously, AUC values of 0.90 were considered as excellent accuracy, 0.80–0.89 as good, 0.70–0.79 as fair, and <0.7 as poor [24,34].

## 3. Results

### 3.1. Study Participants

The study included 23 participants aged between 4–18 years (10 ± 3 years) with physical disabilities (11 of them with CP); weight: 33.2 ± 12.5 kg; height: 129.2 ± 19.9 cm). The characteristics of both groups (whole sample and CP subgroup) are presented in Table 1.

There were no missing data due to errors attributable to accelerometers during the recording or downloading process. Table 2 shows the results obtained for the four proposed physical activity conditions ((i) rest; (ii) comfortable paced walking; (iii) brisk-paced walking; and (iv) fast-paced walking), both in the whole sample and in the CP subgroup (see Supplementary file S1).

### 3.2. Study Objective (i): To Analyze the Accuracy of Evenson Cut-Points Estimating MVPA and SB in Children and Adolescents with Heterogeneous Disabilities Who Can Walk with or without Devices

When examining the ability of the Evenson cut-points to accurately classify intensity, we found that the SB and MVPA were classified with relatively good accuracy. The VM-axis correctly classified 86% of the study participants when they perform SB, whereas the Y-Axis correctly classified 85% when they perform MVPA. However, the percentage of correctly classified children and adolescents with disabilities when they perform MVPA were 88% and 63% using the Y-Axis and the VM counts, respectively. The ROC curve analyses are shown in Table 3.

### 3.3. Study Objective (ii): To Define New Equations to Estimate EE with the GT3X+ in Children and Adolescents with Heterogeneous Disabilities or CP Who Can Walk with or without Devices

**Figure 1:**
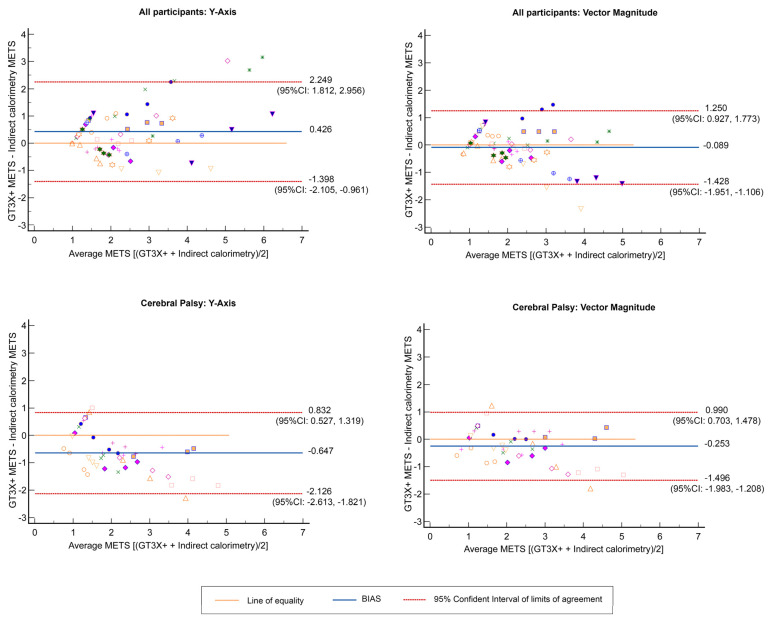
Bland–Altman plots with multiple measurements per individual (energy expenditure (EE), in METs, predicted with GT3X+-EE (METs) determined with indirect calorimetry) by groups.

The best possible equations calculated for the estimation of METs in children and adolescents with heterogeneous disabilities or CP (i.e., the whole sample or CP subgroup) who can walk with or without devices are shown in Table 4. The leave-one-out cross-validation analysis confirmed the coefficients of each variable and the constant in the whole sample and in the CP subgroup. Also, the Bland–Altman plots are shown in Figure 1. For the whole sample, the bias between the differences of EE predicted from the Y-Axis and the indirect calorimetry was 0.426 (LOA: −1.40–2.25) and between the EE predicted from VM activity counts and the indirect calorimetry was −0.089 (LOA: −1.43–1.25). In the CP subgroup, the EE predicted from the Y-Axis and the indirect calorimetry was −0.65 (LOA: −2.13–0.83) and from VM activity counts and the indirect calorimetry was −0.253 (LOA: −1.50–0.99). Heteroscedasticity was not present in the results (*p* > 0.05). 

### 3.4. Study Objective (iii): To Define GT3X+ Cut-Points to Estimate MVPA in Children and Adolescents with CP

Activity cut-points defined from the VM and from the Y-Axis in the CP subgroup are shown in Table 5. Values of the area under the ROC curve, sensitivity, and specificity for the proposed cut-points are shown in Table 5. The MVPA cut-points were ≥702 counts·15 s ^−1^ for VM and ≥360 counts·15 s^−1^ for the Y-Axis. The accuracy to identify MVPA in children and adolescents with CP was excellent regardless of whether it was from the Y-Axis (sensitivity = 92%; specificity = 84%; AUC = 0.907) or the VM cut-point (sensitivity = 93%; specificity = 83%; AUC = 0.900).

## 4. Discussion

The main study findings can be summarized as follows. First, the use of Evenson cut-points with children and adolescents with heterogeneous disabilities who can walk with or without devices correctly classified 86% of the SB cases using the VM activity counts and 88% of the MVPA cases from the Y-Axis activity counts. Second, we defined new equations to predict EE in children and adolescents with heterogeneous disabilities (or CP) who can walk with or without devices. Specifically, the activity counts of VM yielded more accurate values for EE prediction than from the Y-Axis in this population, as well as in analyses conducted specifically in children and adolescents with CP. Third, we also defined specific GT3X+ cut-points for estimating MVPA levels in children and adolescents with CP.

The study conducted by Evenson and colleagues [6], which had a limited sample size (only 33 participants), is one of the conventional references used in analyses of PA and sedentary patterns in children. The cut-points defined by them for the ActiGraph model #AM7164-2.2 have been used with several ActiGraph models (such as with the GT1M, the GT3X, and the GT3X+) and study populations with and without pathology [39]. Despite their extended use acknowledged by the literature, these classical cut-points have not been currently validated in cohorts of children and adolescents with heterogeneous disabilities who can walk with or without devices (i.e., the daily reality of many clinicians). Our results show that, even with such a diverse population, the cut-points established by Evenson and colleagues may be accurate enough to classify SB and MVPA from the GT3X+ counts, which makes them functional and of clinical usefulness for specialists working on a daily basis with children and adolescents exhibiting a wide range of disabilities.

Prediction equations may provide meaningful data about the index of EE from accelerometer counts [40]. Our results show that the use of the VM counts to estimate EE seems to provide a more accurate estimation than the Y-Axis activity counts. This fact agrees with previous results by our research group, in which we reported that the VM from GT3X counts allowed a more accurate EE prediction than the Y axis in young and adult populations [13]. Also, our results here show that the use of specific cut-points for a definite population, as is the case of CP, provides a precise estimation of the performed PA intensity. Clanchy and colleagues, besides reviewing the most commonly used tools to evaluate PA in CP children [24], analyzed the accuracy of the cut-points by Evenson and colleagues with the ActiGraph 7164 in this population. They concluded that the classical cut-points [6] may be used with this ActiGraph model. Moreover, they proposed MVPA-specific cut-points for the ActiGraph 7164 to be used with children and adolescents with CP, which were very similar to those reported by Evenson and colleagues (503 vs. 573 counts·15 s^−1^ or 2012 vs. 2292 counts·min^−1^, respectively). The MVPA cut-points proposed in our study (360 Y-Axis counts·15 s^−1^ or 702 VM counts·15 s^−1^) differ from theirs. Although the protocols and statistical analyses used by Clanchy and colleagues [24] are similar to ours (the same four conditions based on verbal clues to identify PA levels), the accelerometer model is different. In fact, cut-points proposed in other study populations for triaxial ActiGraph accelerometer models are larger than the older uniaxial, omnidirectional, or biaxial models [13].

Other authors assessed the accuracy of the triaxial ActiGraph accelerometer models by estimating PA intensity levels in children and adolescents with CP [22,23]. Although they used more complex analysis data, their results did not provide higher accuracy than our cut-points or than the results by Clanchy and colleagues (2011) using the classical Evenson’s cut-points. Goodlich and colleagues [22] were the first to use machine-learning models to classify PA intensities from the GT3X+ activity counts. Their populations were more motor affected than our children and adolescents with CP because they included only individuals with GMFCS levels III or IV. Also, the standardized activities defined by them compromised other types of motor patterns than ours, such as rest, coloring, overground walking with a mobility aid, wheelchair propulsion, or cycling on a modified tricycle. Moreover, the nature of the PA and the motor impairment of the child may affect the classification accuracy of the cut-points, overestimating SB or light PA because movements without reciprocal leg movements might be not detected by the accelerometer [41]. Furthermore, Trost and colleagues [23] defined a “decision tree” from the GT3X activity counts to estimate PA intensities (sedentary, light PA, and MVPA) in children with CP (GMFCS levels I–III). A decision tree is a pattern recognition (based on a machine learning approach) that categorizes the dependent variable from different values of one or more independent variables. In the case of the study by Trost and colleagues, the model defined intensity-based count thresholds for children and adolescents with CP. To create the model, they defined a design compromising seven standardized activities trials: supine rest, seated handwriting, two housework activities (such as wiping down a countertop and walking or doing the laundry), and three walking intensities (comfortable, brisk, and fast walk). Trost and colleagues indicated that their Y-Axis and VM count models provided congruent classification accuracy in identifying MVPA (with a sensitivity of 79% and 81%; a specificity of 81% and 90%; and an AUC of 0.86 in both, for the Y-Axis and VM models, respectively). These results agree with our results because our cut-points for the Y-Axis and VM counts seem to identify MVPA; however, the specificity shown for our Y-Axis model provided higher values than the VM model (84% vs. 83%, respectively). Intriguingly, Oftedal and colleagues [42] indicated that counts from the VM were more accurate to estimate SB than counts from the Y-Axis in children with CP; in the same line, Keawutan and colleagues [41] concluded that the cut-points derived from the VM were valid to measure SB in children with CP aged from four to five years. Future studies should assess the models provided by Trost and colleagues [23], by Goodlich and colleagues [22], and by us in an independent population. However, although complex, these analyses [22,23] may be useful to estimate PA in children and adolescents with severe motor impairments because their patterns may be modified [22,23,43].

### Limitations

Our study has two major caveats. First, all the PA protocols (i.e., treadmill walking/running) were performed in a laboratory setting instead of being implemented in living conditions. The precision of equations and cut-points should be assessed under free-living conditions with an independent sample of children and adolescents [44]. In addition, as the subgroup with CP only included 11 participants, our results should be considered as preliminary results. Future studies should be carried out to assess the generalizability of the equations and cut-points to free-life settings in a larger sample size.

## 5. Conclusions

**Figure 2:**
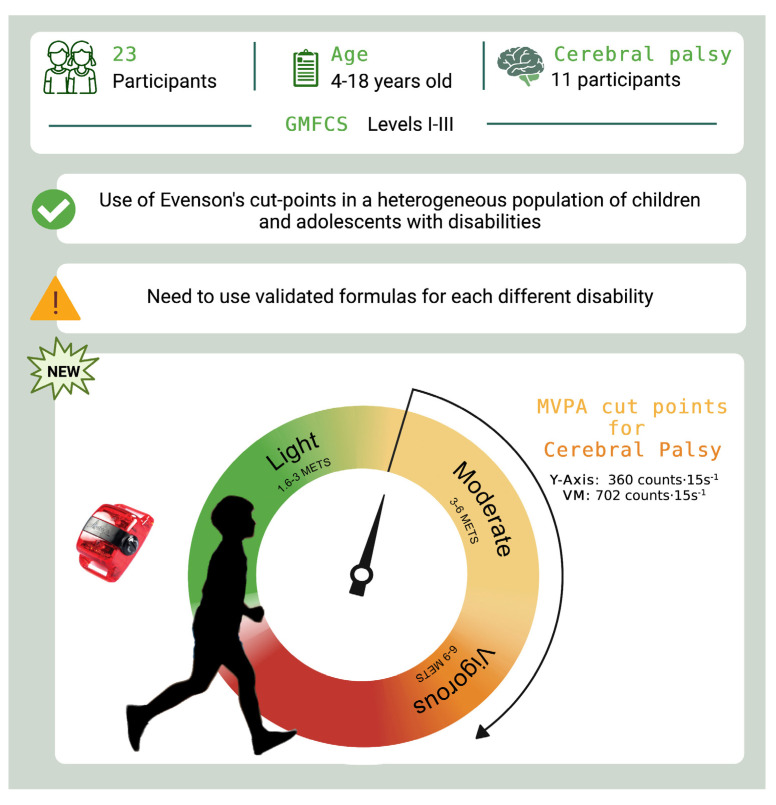
Graphical abstract.

The use of specific cut-points for ActiGraph GT3X+ seems to be accurate to estimate PA levels in children and adolescents with disabilities and, particularly, in those with CP, at least under laboratory conditions (see graphical abstract, Figure 2).

## Figures and Tables

**Table 1:** Descriptive characteristics of the participants.

Outcome	All (*n* = 23)	Cerebral Palsy (*n* = 11)
Age (yr), mean (SD)	10 ± 3 (5–18)	11 ± 4 (5–18)
Gender, *n* females (%)	10 (44%)	4 (36%)
Height (cm), mean (SD)	129.2 ± 19.9	135.5 ± 17.9
Weight (kg), mean (SD)	33.2 ± 12.5	34.6 ± 12.3
GMFCS, *n* (%)		
Level I	13 (57%)	5 (46%)
Level II	4 (17%)	1 (9%)
Level II	6 (26%)	5 (46%)
Coginitive, *n* (%)		
Average	12 (57%)	7 (33%)
Mild impairment	7 (33%)	2 (18%)
Moderate impairment	4 (19%)	2 (18%)
School type, *n* (%)		
Main stream (included)	19 (90%)	9 (82%)
Main stream (self-contained)	2 (10%)	1 (9%)
Special school	2 (10%)	1 (9%)
Clinical diagnosis, *n* (%)		
Cerebral palsy	11 (48%)	11 (100%)
Prader–Willi syndrome	3 (13%)	-
Williams syndrome	1 (4%)	-
Spina bifida	1 (4%)	-
Diencephalic brain tumor	1 (4%)	-
Autism spectrum disorder	1 (4%)	-
Achondroplasia	1 (4%)	-
Metabolic disease	2 (9%)	-
Congenital malformation of upper limbs	1 (4%)	-

**Table 2:** Descriptive statistics by group and by condition.

	Condition	Speed (km·h^−1^)	HR (bpm)	VO_2_ (L·min^−1^)	METs	Axis Y Counts·15s^−1^	VM Counts·15s^−1^
All (*n* = 23)	Rest	−	91 ± 12	0.26 ± 0.05	1.0 ± 0.0	2 ± 5	12 ± 13
	Comfortable paced walking	1.8 ± 0.6	121 ± 3	0.51 ± 0.19	2.0 ± 0.7	160 ± 130	500 ± 233
	Brisk paced walking	2.7 ± 0.8	128 ± 4	0.60 ± 0.26	2.4 ± 0.9	381 ± 304	754 ± 350
	Fast paced walking	3.4 ± 1.0	134 ± 4	0.71 ± 0.31	2.8 ± 1.0	578 ± 414	989 ± 451
Cerebral palsy (*n* = 11)	Rest	−	90 ± 14	0.24 ± 0.05	1.0 ± 0.0	2 ± 3	11 ± 10
	Comfortable paced walking	1.7 ± 06	127 ± 17	0.58 ± 0.23	2.4 ± 0.9	150 ± 148	477 ± 297
	Brisk paced walking	2.7 ± 0.8	138 ± 19	0.74 ± 0.24	3.0 ± 1.0	440 ± 390	804 ± 450
	Fast paced walking	3.2 ± 0.9	146 ± 18	0.86 ± 0.3	3.5 ± 1.2	647 ± 507	1087 ± 544

Bpm, beats per minute; HR, hear rate; HRR, heart rate reserve; METs, metabolic equivalents of tasks; VM, vector magnitude; VO2, oxygen consumption.

**Table 3:** Accuracy of Evenson’s cut-points to estimate MVPA and SB in children and adolescents with disabilities.

Group	Variable	Sensitivity (%)	Specificity (%)	AUC	Correctly Classified (%)	SE
Sedentary (<25 counts·15 s^−1^)	Y	75	90	0.825	85	0.0435
	VM	59	100	0.797	86	0.0441
Moderate-to-vigorous (≥574 counts·15 s^−1^)	Y	67	91	0.790	88	0.0728
	VM	92	59	0.758	63	0.0500

AUC, area under the curve; VM, vector magnitude; SE, standard error.

**Table 4:** Best possible equations calculated for the estimation of METs for each group.

Group	Axis	Equation	RMSE	*p*-Value
All participants (*n* = 23; 10 girls)	Y	METS = 0.383 + 0.001 · Y-Axis AC + 0.020 · BM + 0.263 · GMFCS	0.62	<0.001
	VM	METS = 0.014 + 0.0004 · VM AC + 0.026 · BM + 0.206 · GMFCS	0.60	<0.001
Cerebral palsy (*n* = 11, 4 girls)	Y	METS = −0.309 + 0.0004 · Y-Axis AC + 0.034· BM + 0.245 · GMFCS	0.61	<0.001
	VM	METS = 2.535 + 0.0004 · VM AC + 0.144 · A − 0.033 · H + 0.037 · BM + 0.190 · GMFCS	0.57	<0.001

A, age (years); AC, activity counts (counts·min^−1^); BM, body mass (kg); GMFCS, Gross Motor Function Classification System (level I, 1; level II, 2; or level III, 3); H, height (cm); RMSE, root sum of squared errors; VM, vector magnitude.

**Table 5:** Cut-points to estimate MVPA in children and adolescents with CP.

	Variable	Cerebral Palsy (*n* = 11)				
		Cut-Point (Counts·15 s^−1^)	Sensitivity (%)	Specificity (%)	AUC (Mean ± Standard Error)	*p-*Value
3 METS (MVPA)	Axis Y	360	92	84	0.907 ± 0.036	**<0.001**
	VM	702	93	83	0.900 ± 0.056	**<0.001**

## Data Availability

Not applicable.

## Supplementary Materials

The following are available online at https://www.mdpi.com/article/10.3390/children10030475/s1, Figure S1.
