# Supplementary figures and images for: Validation and Determination of Physical Activity Intensity GT3X+ Cut-Points in Children and Adolescents with Physical Disabilities: Preliminary Results in a Cerebral Palsy Population
Source: Children (Basel). 2023 Feb 27;10(3):475. doi: 10.3390/children10030475 (PMC10047505; doi:10.3390/children10030475)

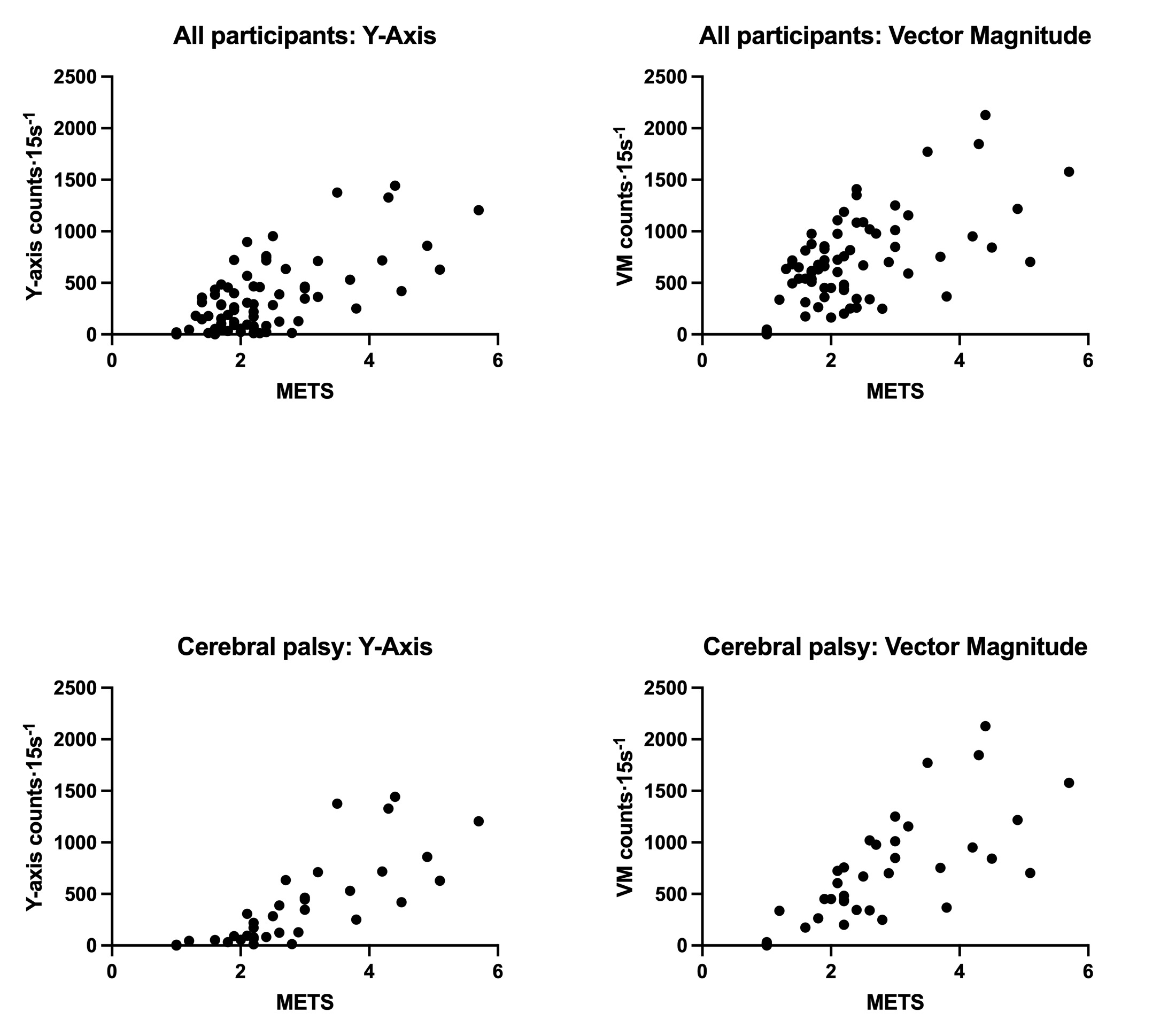

Supplement: Supplementary file 1 [file children-10-00475-s001.zip › children-2134283-supplementary.jpg]
